# CDK6 activity in a recurring convergent kinase network motif

**DOI:** 10.1096/fj.202201344R

**Published:** 2023-03-08

**Authors:** Christina G Gangemi, Rahkesh T. Sabapathy, Harald Janovjak

**Affiliations:** ^1^ Australian Regenerative Medicine Institute (ARMI), Faculty of Medicine, Nursing and Health Sciences Monash University Victoria Clayton/Melbourne Australia; ^2^ European Molecular Biology Laboratory Australia (EMBL Australia) Monash University Victoria Clayton/Melbourne Australia; ^3^ Flinders Health and Medical Research Institute College of Medicine and Public Health, Flinders University South Australia Bedford Park/Adelaide Australia

**Keywords:** convergence, inhibitor, kinase, protein engineering, signaling network

## Abstract

In humans, more than 500 kinases phosphorylate ~15% of all proteins in an emerging phosphorylation network. Convergent local interaction motifs, in which ≥two kinases phosphorylate the same substrate, underlie feedback loops and signal amplification events but have not been systematically analyzed. Here, we first report a network‐wide computational analysis of convergent kinase‐substrate relationships (cKSRs). In experimentally validated phosphorylation sites, we find that cKSRs are common and involve >80% of all human kinases and >24% of all substrates. We show that cKSRs occur over a wide range of stoichiometries, in many instances harnessing co‐expressed kinases from family subgroups. We then experimentally demonstrate for the prototypical convergent CDK4/6 kinase pair how multiple inputs phosphorylate the tumor suppressor retinoblastoma protein (RB) and thereby hamper in situ analysis of the individual kinases. We hypothesize that overexpression of one kinase combined with a CDK4/6 inhibitor can dissect convergence. In breast cancer cells expressing high levels of CDK4, we confirm this hypothesis and develop a high‐throughput compatible assay that quantifies genetically modified CDK6 variants and inhibitors. Collectively, our work reveals the occurrence, topology, and experimental dissection of convergent interactions toward a deeper understanding of kinase networks and functions.

## Graphical abstract

Gangemi *et al.* explored the complex relationships of human kinases and their substrates (top) using a combination of bioinformatics (bottom left) and cell‐based assays (bottom right). They focused on understanding convergent local network motifs in which more than one kinase phosphorylates the same substrate or the same site on a substrate. They found that these motifs are common and can be decoded in the case of the CDK4/6 pair using a combination of kinase inhibition and overexpression.

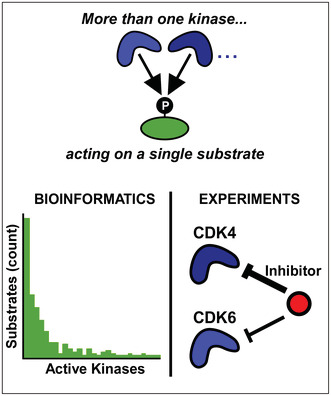

**Abbreviations**
CDK: cyclin‐dependent kinase
cKSRs: convergent kinase‐substrate relationships
HA: hemagglutinin
LY: abemaciclib
MS: mass spectrometry
p18: p18INK4c
PB: palbociclib
ps: photoswitchable
PSP: PhosphoSitePlus
RB: retinoblastoma protein
RI: ribociclib
Ser: serine
Thr: threonine
TK: tyrosine kinase
TPM: transcripts per million
Tyr: tyrosine

## 1 INTRODUCTION

**FIGURE 1:**
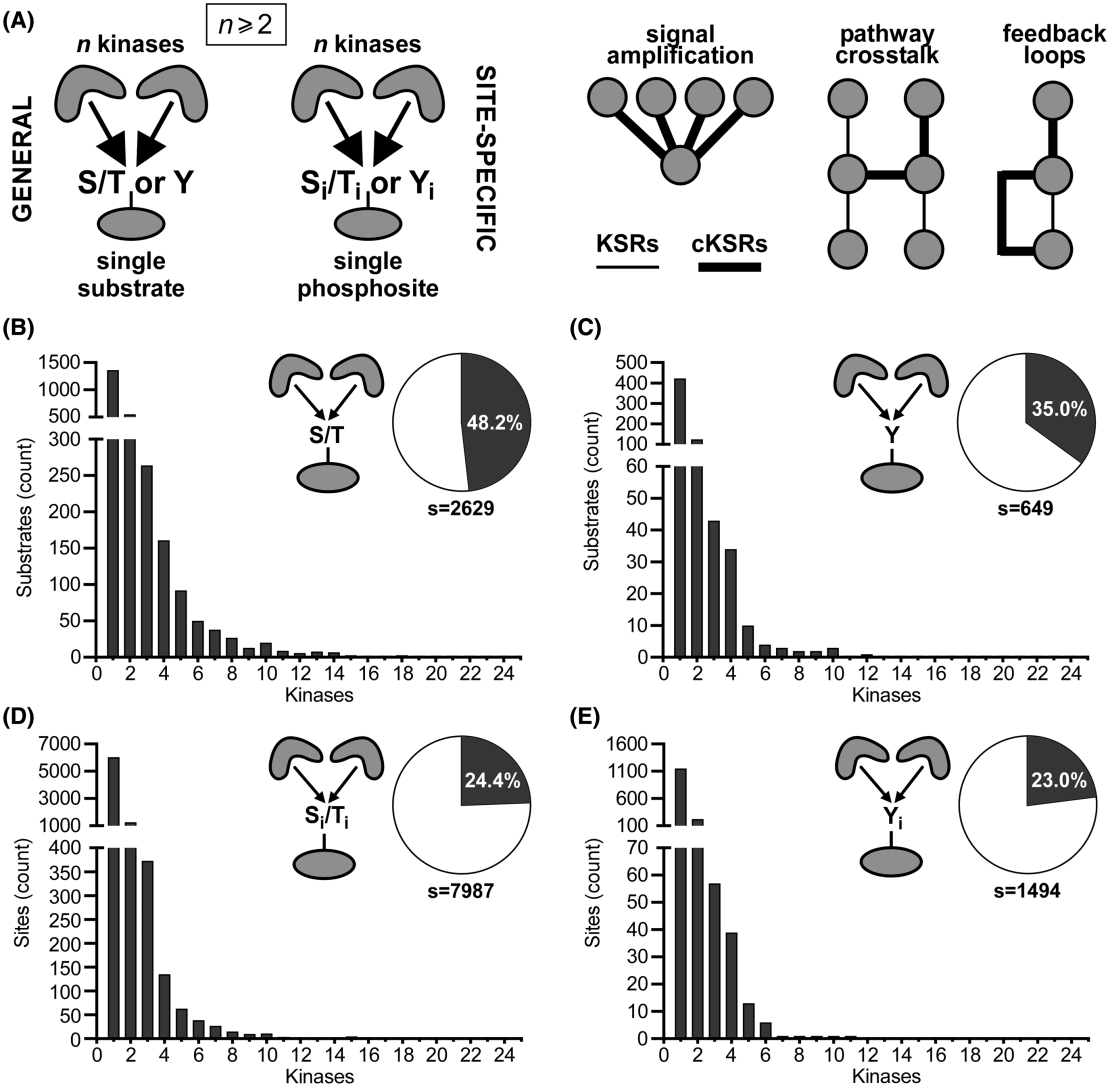
Substrate‐centric analysis of convergent motifs for Ser/Thr and Tyr phosphorylation events. (A) cKSRs are defined as kinase‐substrate interactions in which ≥two kinases phosphorylate a common substrate (general cKSRs) or a common site on a substrate (site‐specific cKSRs). These interactions are involved in, for example, amplification events, pathway crosstalk, and feedback loops. (B, C) Distribution histograms of general cKSRs for Ser/Thr phosphorylation (B) and Tyr phosphorylation (C). Bars indicate how many substrates are phosphorylated by the indicated number of kinases. Pie charts indicate the percentage of substrates that are phosphorylated by ≥two kinases and the total number of analyzed substrates (s). (D, E) Distribution histograms of site‐specific cKSRs for Ser/Thr phosphorylation (D) and Tyr phosphorylation (E). Bars indicate how many sites are phosphorylated by the indicated number of kinases. Pie charts indicated the percentage of sites that are phosphorylated by ≥two kinases and the total number of analyzed sites (s).

Protein phosphorylation is the most common reversible post‐translational modification in eukaryotes and an essential regulator of cell and organism physiology in health and disease. Recent experimental analysis of the phosphoproteome has revealed a large number of human protein phosphorylation sites (e.g., >200 000 sites in large mass spectrometry‐based datasets, or >10 000 sites in datasets of experimentally validated kinase‐substrate interactions).^1^, ^2^, ^3^, ^4^, ^5^ While previous studies of the emerging phosphorylation network focused on global interaction maps or linear/interconnected signaling pathways (reviewed in Refs. [5, 6, 7]), less attention has been devoted to a network‐wide analysis of local motifs.^8^ With ~500 kinases and ~3 000 substrate proteins encoded in the human genome, it is expected that each kinase phosphorylates many sites and that on each substrate protein multiple sites are phosphorylated. Nodes in which multiple kinases converge on one substrate (Figure 1A, left) are likely to occur in the network but their number and topologies have not been systematically investigated. These convergent network motifs are of interest not only because of their involvement in biological functions, including signal amplification, pathway crosstalk, and feedback loops (Figure 1A, right), but also because they can lead to challenges during experimental interrogation. For instance, quantifying the activity of a kinase in situ can be hampered by background phosphorylation of its substrate by additional convergent kinases. Indeed, in well‐known cases of convergence, the presence of multiple inputs has resulted in limited experimental methods for functional analysis (see below).

One prototypical case of phosphorylation convergence centers around the CDK4/6 serine (Ser)/threonine (Thr) kinase pair. CDK4/6 and their associated D‐type cyclins (Cyclin D1/D2/D3) are core components of the cell cycle machinery and regulate commitment to cycle entry.^9^, ^10^ Unbound CDK4/6 are catalytically inactive. Assembled active CDK4/6‐D‐type cyclin complexes are regulated positively (e.g., by the CDK‐activating complex^11^, ^12^), negatively (e.g., by INK4 proteins^10^, ^13^, ^14^), as well as positively or negatively in a context dependent manner (e.g., by KIP proteins^10^, ^13^, ^15^, ^16^). Within the cell cycle, CDK4/6 phosphorylate key regulatory sites on the major growth suppressor RB.^17^, ^18^ RB scavenges E2F transcription factors that are required for cell cycle progression and phosphorylation by various cyclins releases this interaction. While their roles in the cell cycle and in many cases their substrate specificity are overlapping,^19^ CDK4/6 also have non‐overlapping kinase activity‐dependent and ‐independent functions outside of the cell cycle.^20^, ^21^, ^22^, ^23^, ^24^ Not surprisingly, CDK4/6 have received attention as major drivers of cancer and are targeted using small molecule inhibitors.^25^, ^26^ However, the absence of inhibitors that are specific for either kinase and their overlapping activity can challenge experimental analysis.

As shown previously, the presence of either CDK4 or CDK6 is sufficient to phosphorylate RB to levels that prohibit analysis of the activity of the other kinase.^19^, ^27^ As a consequence, CDK4 and CDK6, in particular in the context of RB phosphorylation, are commonly functionally assayed in vitro, either using purified kinases and substrates, such as a recombinant RB fragment,^28^, ^29^, ^30^, ^31^ or after immunoprecipitation.^31^, ^32^ More recently, fluorescent biosensors of CDKs were developed but these require substrate‐specific chemically functionalized polypeptide probes and have not been deployed in living cells.^33^, ^34^, ^35^ In cells, exploration of potential CDK6 substrates was conducted using gel‐based electrophoretic migration shift assays that do not offer high throughput and may not in all cases provide the resolution required to visualize phosphorylation events.^22^ Finally, transcriptional reporter plasmids for signals downstream of RB often exhibit low signal‐to‐noise ratios.^36^, ^37^, ^38^ Collectively, assaying the CDK4/6‐RB‐axis in complex cellular environments and compatible with parallelization is currently challenging. Because of its biological/biomedical significance and these experimental limitations, the CDK4/6 pair represents an interesting test case for experimental convergence analysis.

Here, we first performed a network‐wide analysis of local kinase‐substrate interactions with a focus on cKSRs in experimentally validated phosphorylation sites. We quantified cKSR frequency, topology, and kinase family subgroup distribution. We then hypothesized that kinase overexpression combined with the use of a CDK4/6 inhibitor provides an approach to assay an individual kinase of the pair. Using this strategy, we were able to characterize genetically modified CDK6s and their inhibitors in breast cancer cells expressing high levels of CDK4. Collectively, our work reveals fundamental properties of cKSRs and demonstrates a possible approach to dissect convergence in cellular contexts even in the absence of kinase‐specific inhibitors.

## 2 MATERIALS AND METHODS

### 2.1 Key resources table

**Table:** 

Reagent or Resource	Source	Identifier
*Reagents*		
Abemaciclib (LY, LY2835219)	AdooQ Bioscience	A12989
Ribociclib (RI, LEE011)	Selleckchem	S7440
Palbociclib (PB, PD‐0332991)	MedChemExpress	HY‐50767A
MEM	Gibco	11095080
Penicillin streptomycin	Gibco	15140122
FBS	Gibco	26140079
Insulin, recombinant human zinc solution	Gibco	12585014
PBS	Gibco	20012050
Opti‐MEM	Gibco	31985070
X‐tremeGENE HP DNA Transfection Reagent	Roche	6366236001
Cell Lysis Buffer (10x)	Cell Signaling Technology	9803S
Glycerol	Sigma‐Aldrich	G5516‐500ML
SDS	Sigma‐Aldrich	L3771‐500G
Bromophenol blue	Sigma‐Aldrich	B0126‐25G
β‐mercaptoethanol	Sigma‐Aldrich	M3148‐25ML
20x Bolt MOPS SDS Running Buffer	Invitrogen	B0001
Bolt 4%–12% Bis‐Tris Plus Gels 15‐well	Invitrogen	NW04125BOX
Bolt 4%–12% Bis‐Tris Plus Gels 10‐well	Invitrogen	NW04120BOX
Immun‐Blot PVDF Membrane	Bio‐Rad	1620177
BSA	Sigma‐Aldrich	A7906‐50G
Tween 20	Sigma‐Aldrich	P1379
Clarity Western ECL Substrate	Bio‐Rad	1705060
*Antibodies*		
DYKDDDDK Tag (D6W5B) Rabbit mAb (FLAG‐Tag)	Cell Signaling Technology	14793
GAPDH (D16H11) XP Rabbit mAb	Cell Signaling Technology	5174
HA‐Tag (C29F4) Rabbit mAb	Cell Signaling Technology	3724
Phospho‐RB (Ser780) (D59B7) Rabbit mAb	Cell Signaling Technology	8180
β‐tubulin (9F3) Rabbit mAb	Cell Signaling Technology	2128
CDK4 (D9G3E) Rabbit mAb	Cell Signaling Technology	12790
RB (4H1) Mouse mAb	Cell Signaling Technology	9309
Goat Anti‐Mouse IgG (H + L)‐HRP Conjugate	Bio‐Rad	1721011
Goat Anti‐Rabbit IgG (H + L)‐HRP Conjugate	Bio‐Rad	1706515
*Kits*		
AlphaLISA SureFire Ultra p‐RB (Ser780)	PerkinElmer	ALSU‐PRB‐C
AlphaLISA SureFire Ultra RB Total	PerkinElmer	ALSU‐TRB‐A
*Cells*		
MCF‐7	American Type Culture Collection (ATCC)	HTB‐22
*Software*		
cKSR analysis formulas/filters	This paper	http://github.com/janovjaklab/convergent_kinases
Co‐expression analysis code	This paper	http://github.com/janovjaklab/convergent_kinases

### 2.2 KSR analysis

The KSR analysis was based on a comprehensive set of experimentally determined phosphorylation sites deposited in PhosphoSitePlus (PSP).^4^ PSP is extensive and continuously manually curated and contains sites for which the phosphorylating kinase is known. Phosphorylation sites were downloaded (http://www.phosphosite.org, v6.6.0.2, February 2022) and the complete set of sites was processed to obtain cKSRs using custom macros and formulas (see below for software availability). A further dataset was provided by PSP/Cell Signaling Technology, Inc., upon request (December 2022) that contained information about the number of experimental reports for each KSR. KSRs with at least two independent reports were included in the analysis shown in Figures S1 and S3.

The first analysis was substrate‐centric. This analysis was performed for Ser/Thr and tyrosine (Tyr) phosphorylation events separately and by considering either *general* cKSRs (i.e., ≥two kinases phosphorylate the same substrate but not necessarily at the same site) or *site‐specific* cKSRs (i.e., ≥two kinases phosphorylate the same substrate at the same phosphorylation site). First, PSP sites were limited to human substrate entries. Second, to eliminate duplicate pairings of kinases and substrates/sites for accurate counting, fingerprints were calculated that combined the kinase and substrate/site name (e.g., “CDK4:RB” or “CDK4:RB:S780”). In a third step, the fingerprints were used to identify unique substrates/sites. In a fourth step and for each unique substrate/site, the number of active kinases (i.e., of those kinases phosphorylating this substrate/site) was counted and their names compiled. For general cKSRs, these unique lists are given in Tables S1 and S2 (Ser/Thr and Tyr phosphorylation, respectively). For site‐specific cKSRs, these unique lists are given in Tables S3 and S4 (Ser/Thr and Tyr phosphorylation, respectively). Finally, the occurrence of each possible cKSR of K:1 topology (where K denotes the number of kinases and is between 1 and 50, and 1 denotes the substrate/site) was counted. This analysis also reported the total number of substrates/sites involved in cKSRs.

The second analysis was kinase‐centric. This analysis was performed for Ser/Thr and Tyr kinases separately. Above kinase‐substrate pairings were reanalyzed to produce filtered lists of kinases. The main applied filter was to include only those kinases which phosphorylate substrates that are subject to general or site‐specific cKSRs (Tables S5–S8). This list was then further used to retrieve kinase‐specific information, such as the number of substrates that is phosphorylated by each kinase.

The utilized macros and formulas are available online in a publicly accessible format (https://github.com/janovjaklab/convergent_kinases).

### 2.3 Kinase family subgroup analysis

Lists containing the kinases that phosphorylate each substrate/site were generated (as in Tables S1–S4). For each kinase, subgroup information was obtained from KinHub (http://kinhub.org, column: group) after correcting, where necessary and in a small number of cases, kinase nomenclature using the nomenclature list of KinHub. For each set of kinases that act on a specific substrate/site, the occurrence of members of the canonical kinase family subgroups AGC, CAMK, CK1, CMGC, RGC, STE, TK, TKL as well as atypical and “other”^39^ was counted and this information encoded in a numeric string. These strings were then analyzed to reveal the number of substrates/sites that are phosphorylated by kinases from multiple family subgroups and the number of substrates/sites that are phosphorylated by ≥two kinases from at least one family subgroup.

### 2.4 Identification of reciprocal phosphorylation loops

Reciprocal phosphorylation loops between two kinases were identified in the general cKSR dataset as follows: First, lists were compiled of all kinases (labeled “A” in Figure S2) that are phosphorylated by multiple kinases, for example, by an input kinase and a kinase in the reciprocal loop. Second, for each of these kinases lists of its substrates were compiled. Third, these substrate lists were compared to lists of kinases that phosphorylate kinase A to identify kinases B_1_‐B_n_. Kinase A‐B pairs are then compiled in Table S13. For each identified phosphorylation loop, the Web Of Science database (Clarivate) was searched with keyword combinations that include the kinases and “feedback loop” or “reciprocal.” Occurrence of prior mention of the reciprocal interactions is also indicated in Table S13.

### 2.5 Co‐expression analysis: Transcriptomics

The first co‐expression analysis was based on an analysis of 1393 cancer cell line transcriptomes collected by the cancer cell line encyclopedia initiative.^40^, ^41^ Expression data of all protein‐coding genes in the log_2_ (TPM + 1) format (where +1 denotes a pseudocount) were downloaded (http://sites.broadinstitute.org/ccle/, http://depmap.org/portal/download/, v22Q1, April 2022) and further processed using custom macros and formulas. This analysis was performed for Ser/Thr and Tyr phosphorylation events separately and by considering either general or site‐specific cKSRs. First, protein names in the expression data were cropped to only contain short HGNC compliant names. Second, the TPM values were converted into a binary format (1: expressed, 0: not expressed) using specified TPM thresholds (5, 10, or 20). Third, lists containing the kinases that phosphorylate each substrate/site were generated (as in Tables S1–S4). Where necessary and in a small number of cases, kinase nomenclature was corrected using the nomenclature list of KinHub (http://kinhub.org). Fourth, for each group of kinases that act on a substrate/site, the number of cell lines was counted in which ≥two convergent kinases are co‐expressed. The percentage was defined as the co‐expression score and is compiled in Tables S9–S12. The utilized macros and formulas are available online in a publicly accessible format (https://github.com/janovjaklab/convergent_kinases).

### 2.6 Co‐expression analysis: Proteomics

The second co‐expression analysis was based on analysis of multiplexed MS data of 375 cancer cell lines.^42^ Expression data were downloaded (https://gygi.hms.harvard.edu/publications/ccle.html, November 2022) and processed using the four steps as for the transcriptomic data above. In the second step (thresholding), a median threshold was applied to identify cell lines that express the protein of interest. This was required as MS data were normalized by reference “bridge” samples and, unlike transcriptomic data in units of TPM, are not absolute values. As for the transcriptomic data, co‐expression analysis was performed for Ser/Thr and Tyr phosphorylation events separately and either general or site‐specific cKSRs. The co‐expression score (defined as for transcriptomics) are summarized in Tables S9–S12.

### 2.7 Reagents

Reagents utilized are listed in the Key Resources Table. Stock solutions for CDK4/6 inhibitors were prepared by dissolving in dimethyl sulfoxide (DMSO) to the following concentrations: abemaciclib (LY, 2 mM), ribociclib (RI, 10 mM), and palbociclib (PB, 5 mM). Inhibitors were further diluted in cell culture media before being added to cells and equivalent amounts of DMSO were added to control wells.

### 2.8 Vectors and constructs

All genes were ordered as synthetic genes (gBlocks, Integrated DNA Technologies) and designed to contain an N‐terminal HA‐tag (YPYDVPDYA), with the exception of Cyclin D3 that contained an N‐terminal FLAG‐tag (DYKDDDDK). gBlocks were amplified in polymerase chain reactions using oligonucleotides with restriction site overhangs and digested with the corresponding restriction enzymes. These were NotI and BamHI for wild‐type (WT) CDK6 and p18^INK4c^, Xhol and BamHI for Cyclin D3, NotI and BstEII for CDK6 with N‐terminal pdDronpa1 (N‐term pdD1), and BstEII and BamHI for CDK6 with internal loop pdDronpa1 (loop pdD1) (New England Biolabs). pcDNA3.1(‐) vector (Thermo Fisher Scientific) was digested with the corresponding enzymes and ligated with amplified genes using T4 DNA ligase (Promega).

Single residue mutations were introduced in WT CDK6 using overlapping oligonucleotides designed in PrimerX (https://www.bioinformatics.org/primerx/) using the QuikChange option. Circular PCRs were performed with a high‐fidelity polymerase (Q5, NEB) followed by digestion with DpnI restriction enzyme (New England Biolabs).

All genes were verified using Sanger sequencing (MicroMon, Monash University). The sequences of the engineered CDK6 fusion proteins are summarized in Table S14.

### 2.9 Cell culture

Human breast cancer MCF‐7 cells (ATCC, HTB‐22) were kindly provided by Antonella Papa (Monash University) and cultured in minimum essential medium (MEM, Gibco) in a humidified incubator with 5% CO_2_ atmosphere at 37°C. Medium was supplemented with 10% FBS, 0.1 mg/mL human recombinant insulin, 100 U/mL penicillin, and 0.1 mg/mL streptomycin (Gibco). Transfections were conducted by seeding 500 000 cells per well in 6‐well plates for immunoblotting experiments or 18 000 cells in 96‐well plates for energy transfer‐based (AlphaLISA) assays. Twenty‐four hours after seeding, cells were transfected with plasmid DNA using X‐tremeGENE HP DNA transfection reagent (Roche) as per the manufacturer's protocol using a total vector amount of 2 μg (6‐well plates) or 0.1 μg (96‐well plates). A 3:1 ratio of transfection reagent to DNA (6 μL:2 μg for 6‐well or 0.3 μL:0.1 μg for 96‐well plates) was used. Single construct controls were supplemented with empty pcDNA3.1(−) vector and for co‐expression plasmids were added at a ratio of 1:1 or 1:1:1. Mock transfections contained empty pcDNA3.1(−) vector only. After 24 h, the culture medium was replaced and CDK4/6 inhibitor/s added.

### 2.10 Immunoblotting

After 24 h of inhibitor treatment, cells were transferred onto ice and washed with cold 1x phosphate‐buffered saline (PBS). Five hundred microliter of 1x cell lysis buffer (Cell Signaling Technology) supplemented with Complete EDTA‐free Protease Inhibitor Cocktail (Roche) was added for cell lysis (no phosphatase inhibitors were added). Lysates were collected and sonicated for 10 s, followed by incubation at 4°C with constant shaking (2000 rpm) for 30 min. Samples were then centrifuged for 20 min at 4°C before adding 4x Laemmli loading buffer (40% glycerol, 240 mM Tris/HCL, pH 6.8, 8% SDS, 0.04% bromophenol blue and 5% β‐mercaptoethanol) and denaturation at 95°C for 5 min. Proteins were separated using Bolt 4%–12% Bis‐Tris Plus Gels (Invitrogen) run at 140 V for 60 min before being transferred onto PVDF membranes (100 V for 100 min) (Bio‐Rad). Membranes were blocked in 1x TBST (1x Tris‐buffered saline, 0.1% Tween) containing 5% skim milk powder for >1 h at room temperature (rotating). Membranes were then probed with indicated primary antibodies (diluted in either 5% skim milk powder or bovine serum albumin in 1x TBST) overnight while rotating at 4°C. Corresponding mouse or rabbit horseradish peroxidase‐conjugated secondary antibodies (diluted 1:10 000 in 1x TBST) were added for 1 h at room temperature (Bio‐Rad). Chemiluminescence was detected by adding Clarity Western ECL Substrate (1:1 of luminol and peroxide solutions) in a ChemiDoc Touch Imaging System (Bio‐Rad).

### 2.11 Energy transfer‐based assays

AlphaLISA SureFire Ultra assay kits (PerkinElmer) were used in a two‐plate two‐incubation assay protocol for adherent cells according to manufacturer's instructions. Briefly, cells were washed with 100 μL of PBS per well, followed by addition of 100 μL of 1x lysis buffer and incubated for 10 min on a plate shaker at 350 rpm. Lysate was transferred to light gray 384‐well untreated AlphaPlates (PerkinElmer) by adding 10 μL per well. Both kits were used to run both assays in parallel on the same lysates from each condition. Five microliter of acceptor mix was added per well and incubated for 1 h in the dark followed by addition of 5 μL of donor mix (under low light conditions) and incubated for a further hour in the dark. Wells were then read using a PHERAstar plate reader (BMG). Each condition was assayed in triplicates per kit.

### 2.12 Data analysis and statistics

Densitometry analysis was performed on immunoblots using ImageLab 6.1 software (Bio‐Rad). Kinase‐substrate topology analysis was performed using equations and filters in Microsoft Excel. Reciprocal interaction analysis, kinase family analysis, and co‐expression analysis were performed using macros written in C in Igor Pro 6.22 (Wavemetrics). Data for Figure S10 were prepared in UniProt (http://www.uniprot.org, alignment of human CDK5 and CDK6) and PyMOL 2.5 (Schrödinger; protein structures: PDB‐ID 1JOW for CDK6 and 1H4L for CDK5). All other data were analyzed using Prism (9.0, GraphPad). Statistical significance of results from immunoblotting and energy transfer‐based assays was determined by one‐way ANOVA statistical test and either the Dunnett's method or Tukey's method to correct for multiple comparisons. Dunnett's method correction was applied when comparing conditions to mock or LY‐treated mock controls. Tukey's method correction was applied when performing multiple comparison between conditions. Only statistically significant comparisons (*p* ≤ .05) are indicated in the figures and captions.

## 3 RESULTS

### 3.1 Convergent motifs are abundant in Ser/Thr and Tyr phosphorylation

We systematically analyzed convergent phosphorylation motifs. As the data source we utilized the comprehensive PSP repository of experimentally determined phosphorylation sites.^4^ >21 000 phosphorylation sites are deposited in PSP across all species but human substrates are dominant (accounting for 13 916 or 64% of all sites). We limited our analysis to these human phosphorylation sites and separated Ser/Thr from Tyr kinase reactions. Using a fingerprinting approach, we identified unique substrates and for each substrate compiled the phosphorylating kinases (Tables S1–S4). We found that convergent motifs are common both in Ser/Thr and Tyr phosphorylation. Of the 2629 substrates of 350 Ser/Thr kinases, 1268 (48%) are phosphorylated by more than one kinase (Figure 1B). Similarly, in the case of Tyr phosphorylation by 129 kinases, 227 (35%) of 649 substrates are phosphorylated by more than one kinase (Figure 1C). We next determined cKSR topologies expressed as *K*:1, where *K* denotes the number of kinases and 1 denotes the substrate. We found that 21%, 10%, and 6.1% of substrates are phosphorylated by two, three, or four Ser/Thr kinases, and the remaining substrates by varying kinase numbers (Figure 1B). We also found that 19%, 6.6%, and 5.2% of substrates are phosphorylated by two, three, or four Tyr kinases, and the remaining substrates by varying kinase numbers (Figure 1C). Thus, cKSRs are not only generally abundant but also occur with broad topologies that are somewhat comparable between Ser/Thr and Tyr phosphorylation.

The analysis conducted so far depicted general cKSRs in which ≥two kinases phosphorylate the same substrate but not necessarily at the same site. Thus, we went on to extend the analysis to site‐specific cKSRs in which the same site is phosphorylated. Also in this case, we observed common convergent motifs in that 1946 (24%) of 7987 Ser/Thr sites or 344 (23%) of 1494 Tyr sites were phosphorylated by ≥two kinases (Figure 1D,E). As for the general cKSRs, a wide range of topologies was observed. We found that 15%, 4.7%, and 1.7% of substrates are phosphorylated by two, three, or four Ser/Thr kinases, and 15%, 3.8%, and 2.6% of substrates phosphorylated by two, three, or four Tyr kinases (Figure 1D,E). Collectively, these results indicate that diverse cKSRs comprise a significant fraction of the human phosphorylation landscape as mapped in the PSP. We repeated this analysis considering only phosphosites that were identified in multiple independent studies and obtained similar results (e.g., 41% and 26% of Ser/Thr and Tyr kinase substrates are phosphorylated by more than one kinase; Figure S1). Overall, these results serve as the fundamental dataset that allows delineating key substrates onto which kinases converge and allows identifying known input kinases for a specific site (Tables S1–S4).

In an exemplary analysis, we queried the dataset for the presence of reciprocal phosphorylation loops in which the substrate of a kinase is able to phosphorylate that kinase (rendering latter kinase into a cKSR substrate in a loop; see Figure S2 for a schematic of this motif). In the general cKSR dataset, we identified interactions between 33 kinases in 31 unique reciprocal loops (Table S13). The majority of these interactions (41 of 62) were Ser/Thr phosphorylation events and included well known feedback loops, such as those upstream the MAPK/ERK pathway and also those in this pathway (e.g., between MEK1 and ERK1). However, the observation that ~half of the identified reciprocal interactions have not been described in the literature previously (Table S13) demonstrates the potential of cKSR data to identify network components with specific topologies for further analysis.

### 3.2 Most kinases phosphorylate cKSR substrates

**FIGURE 2:**
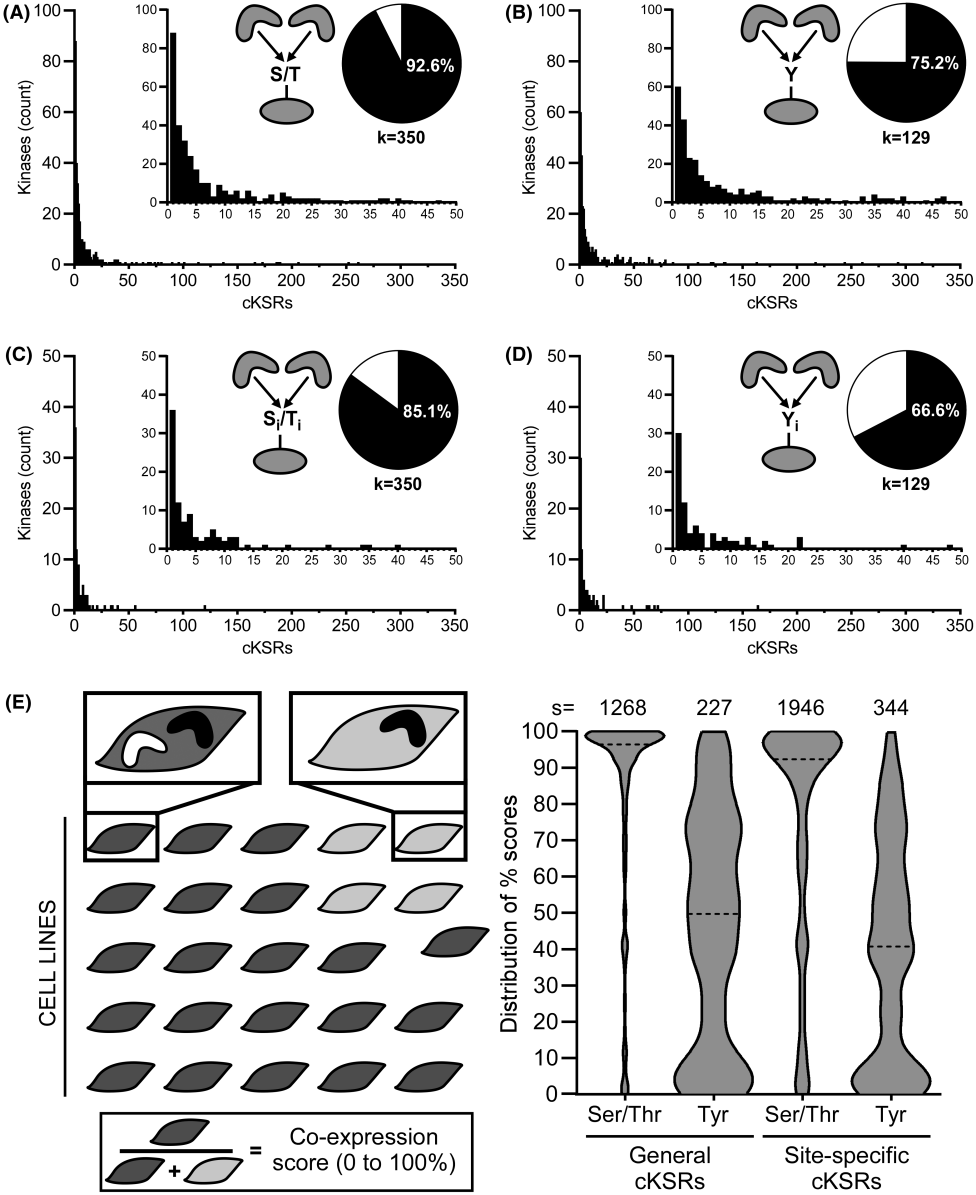
Kinase‐centric analysis of convergent motifs for Ser/Thr and Tyr phosphorylation events. (A, B) Distribution histograms for Ser/Thr kinases (B) and Tyr kinases (C) in general cKSRs. Bars indicate how many kinases phosphorylate the indicated number of substrates. Pie charts indicate the percentage of kinases that participate in general cKSRs and the total number of kinases (k). (C, D) Distribution histograms for Ser/Thr kinases (B) and Tyr kinases (C) in site‐specific cKSRs. Bars indicate how many kinases phosphorylate the indicated number of sites. Pie charts indicate the percentage of kinases that participate in site‐specific cKSRs and the total number of kinases (k). (E) Co‐expression analysis of converging kinases in cancer cell lines. For each group of kinases that phosphorylate the same substrate or site, the number of 1393 cancer cell lines that co‐express these kinases was counted. From this, a co‐expression score was calculated as the ratio of cells with (dark gray) and without (light gray) co‐expression. Violin plots summarize the distribution of these scores for the analyzed number of substrates or sites (s) (dashed line: median; TPM threshold: 10).

We next investigated how many kinases participate in cKSRs and whether some interaction stoichiometries are more common than others (e.g., are few kinases acting on many substrates, or are many kinases acting on few substrates?). We defined the kinases of interest as those that act on substrates that are phosphorylated by ≥two kinases. We found that this criterion applied to a large fraction of human kinases (88% or 421 of 479 kinases listed in the PSP) (Figure 2A–D). More specifically, of 350 Ser/Thr kinases, 324 or 298 (93% or 85%) were identified to act on general or site‐specific cKSR substrates (Figure 2A,C, Tables S5 and S7). For the 129 Tyr kinases, fractions were somewhat lower but still indicated prominence (75% or 67% were associated with general or site‐specific cKSR substrates; Figure 2B,D, Tables S6 and S8). We also found that kinase stoichiometries were broadly distributed. Of the identified kinases, most phosphorylated a small number of substrates while a small number of kinases acted on many substrates (Figure 2A–D). We repeated this analysis considering only phosphosites that were identified in multiple independent studies and obtained similar results (e.g., 93% of Ser/Thr kinases and 76% of Tyr kinases were identified to act on general cKSR substrates; Figure S3). Collectively, these results indicate that convergent motifs involve a large number of kinases through complex interactions. They allow identifying selected kinases that drive the largest number of phosphorylation events (e.g., the major signaling regulatory Ser/Thr and Tyr kinases ERK2 and SRC; Tables S7 and S8).

We extended this analysis to query if kinases from multiple family subgroups converge on the same substrate or site. Our analysis focused on the major kinase family subgroups AGC, CAMK, CK1, CMGC, RGC, STE, TK, TKL as well as atypical and “other”.^39^ We found that multi‐family phosphorylation is common. In the case of general cKSRs, 891 (or 70%) of 1268 Ser/Thr substrates, or 27 (or 12%) of 227 Tyr substrates were phosphorylated by kinases of ≥two families (Figure S4A,B). In the case of site‐specific cKSRs, 791 (or 41%) of 1946 Ser/Thr sites, or 20 (or 5.8%) of 344 Tyr sites were phosphorylated by kinase of ≥two families (Figure S4C,D). The lower abundance of Tyr phosphorylation by multiple families needs to be interpreted with caution as it is likely due to the concentration of >90% of Tyr kinases in the TK (tyrosine kinase) group. We further investigated if multiple kinases from the same family subgroup participate in cKSRs. Strikingly, we found that this indeed applies to sizeable fractions of substrates (73% for Ser/Thr phosphorylation and 95% for Tyr phosphorylation) and sites (78% for Ser/Thr phosphorylation and 98% for Tyr phosphorylation) (Figure S5). These observations motivated the experimental exploration of strategies to dissect the activity of closely related kinases on single substrates (see below).

Finally, we also asked if these kinases are co‐expressed to relate to biological effects of cKSRs. We analyzed RNA‐sequencing data from 1393 human cell lines deposited in the DepMap portal and cancer cell line encyclopedia.^40^ Similar to above, we compiled lists of active kinases (i.e., of those kinases phosphorylating this substrate/site) for either each cKSR substrate or each cKSR site. We then examined expression levels of these kinases (TPM ≥ 10 in Figure 2E, and also TPM ≥ 5 and 20 in Figure S6, all values are given in Tables S9–S12). As a score for co‐expression in each kinase group, we quantified in how many of the 1393 cell lines at least two convergent kinases are expressed (Figure 2E, left). We found that cases of co‐expression are generally frequent (Figure 2E, right). On average and for TPM ≥ 10, the identified Ser/Thr kinase groups are co‐expressed in >1000 (or >70%) of the analyzed cell lines. Furthermore, only a small number of groups (<4%) were co‐expressed more rarely (in <2.5% of cell lines). For Tyr kinases, on average the groups were co‐expressed in >500 (or >38%) of the analyzed cell lines with <11% of the groups co‐expressed more rarely (in <2.5% of cell lines). Albeit co‐expression is generally more prominent for Ser/Thr compared to Tyr kinases, these data collectively indicate that not only are large numbers of kinases participants in cKSRs but also that these kinases are in many cases co‐expressed. Prominent co‐expression was confirmed in cancer cell proteomics data (Figure S7) where kinase groups were on average co‐expressed in 40% (Ser/Thr) and 25% (Tyr) of analyzed cell lines. The co‐expression data based on transcriptomics and proteomics tabulated in Tables S9–S12 allow identifying for specific kinases cellular contexts with and without convergence.

### 3.3 Consequences of cKSRs in a prototypical kinase pair

The above analysis points to a significant occurrence of cKSRs. This promoted us to explore the consequences of convergence and to develop a strategy to dissect it for a prototypical kinase pair of the same family where specific inhibitors are unavailable. This kinase pair consists of CDK4 and CDK6, two cyclin‐dependent kinases that act on the major cell cycle regulator protein RB. We selected the human breast cancer cell line MCF‐7 as the main experimental system. This cell type is known to express high levels of CDK4,^13^, ^27^ which we confirmed by immunoblotting (Figure S8) and in the CCLE transcriptomic data (TPM_CDK4_: 170), but very low CDK6 levels (generally not detectable on immunoblots,^27^, ^43^, ^44^ TPM_CDK6_: 0.39^41^). For this reason, this cell type offers the possibility to supplement one kinase with the other.

**FIGURE 3:**
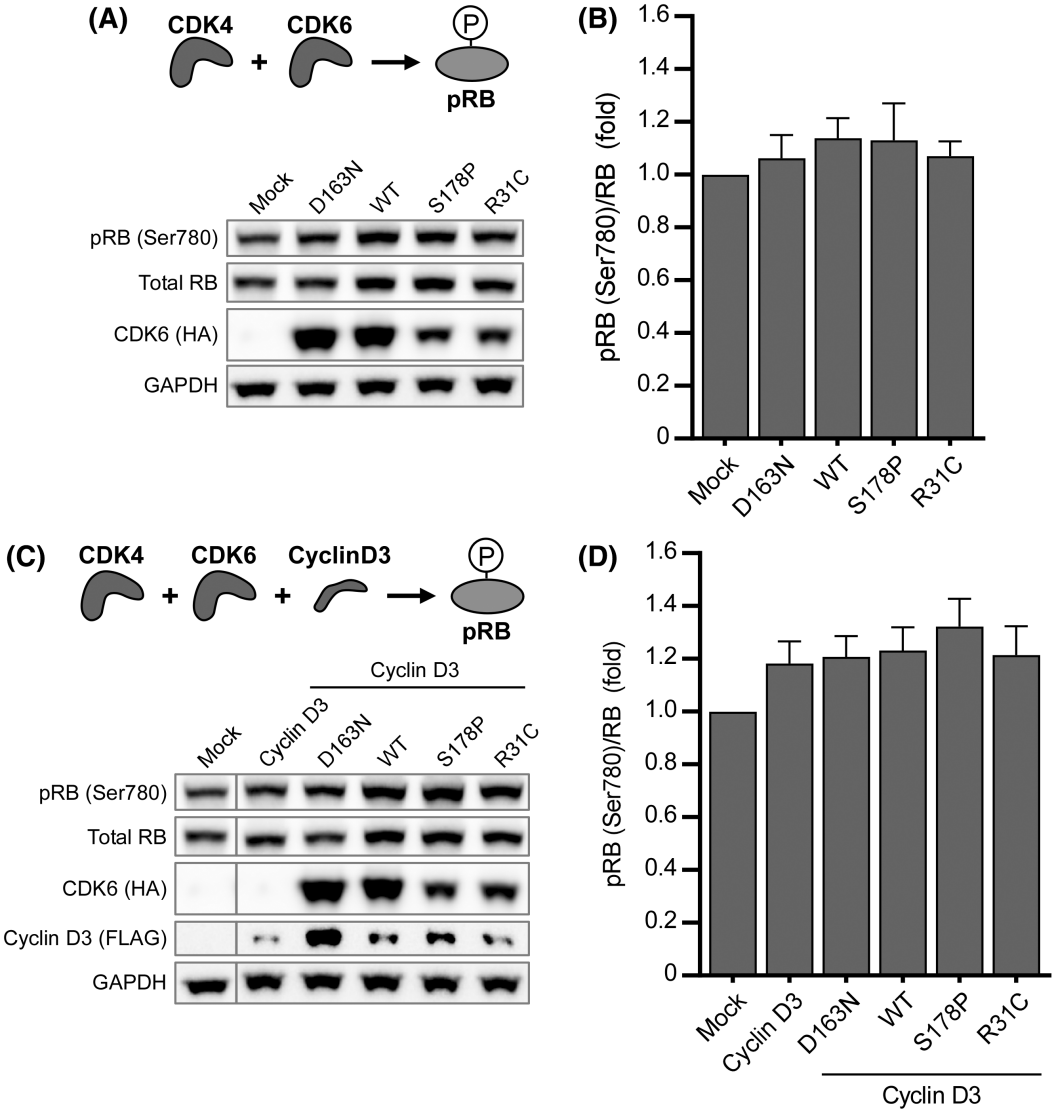
Overexpression of CDK6 and variants does not increase pRB levels. (A) Immunoblot analysis of pRB in MCF‐7 cells expressing CDK6 and its variants. (B) Densitometry analysis of data shown in (A) expressed as a ratio of pRB to total RB. (C) Immunoblot analysis of pRB in MCF‐7 cells expressing CDK6 and its variants when co‐transfected with Cyclin D3. (D) Densitometry analysis of data shown (C) expressed as a ratio of pRB to total RB. (A and C) Representative experiments are shown. (B and D) *n* = 3. Data are mean ± SEM. One‐way ANOVA test with Dunnett's method correction compared with mock‐transfected control: No significance was detected.

Untreated MCF‐7 cells exhibit high levels of phosphorylated RB (pRB, Ser780) attributable to CDK4 (Figure 3A,B).^27^, ^43^ We tested whether overexpression of CDK6 can elevate pRB levels but observed only a minimal and not statistically significant effect (~1.1‐fold) in transfected cells. We also examined CDK6 variants harboring two activating mutations, S178P^32^ or R31C.^45^ The S178P mutant results in the cyclin‐independent activation of CDK6 and increased cyclin‐dependent activity in vitro,^32^ while the R31C mutation prevents the binding of INK4 protein inhibitors resulting in sustained kinase activity.^45^ Both activating mutations, however, did not result in higher pRB levels compared to WT CDK6 (Figure 3A,B). To test the possibility that D‐type cyclins are limiting any effect of overexpressed CDK6,^13^, ^46^ we co‐transfected Cyclin D3. This D‐type cyclin was chosen over D1 and D2 as it is known to promote high levels of kinase activity when in complex specifically with CDK6.^13^, ^46^ Cyclin D3 overexpression resulted in minor increases in pRB levels (~1.2‐fold, not statistically significant) (Figure 3C,D). A similar result was observed for CDK6‐S178P and ‐R31C co‐transfected with Cyclin D3 (~1.3‐ and 1.2‐fold, resp.) (Figure 3C,D). Collectively, these results indicate that overexpression of CDK6 in cells that exhibit high background activity of the convergent CDK4 does not alter pRB levels.

### 3.4 CDK4/6 inhibition to isolate CDK6 activity

**FIGURE 4:**
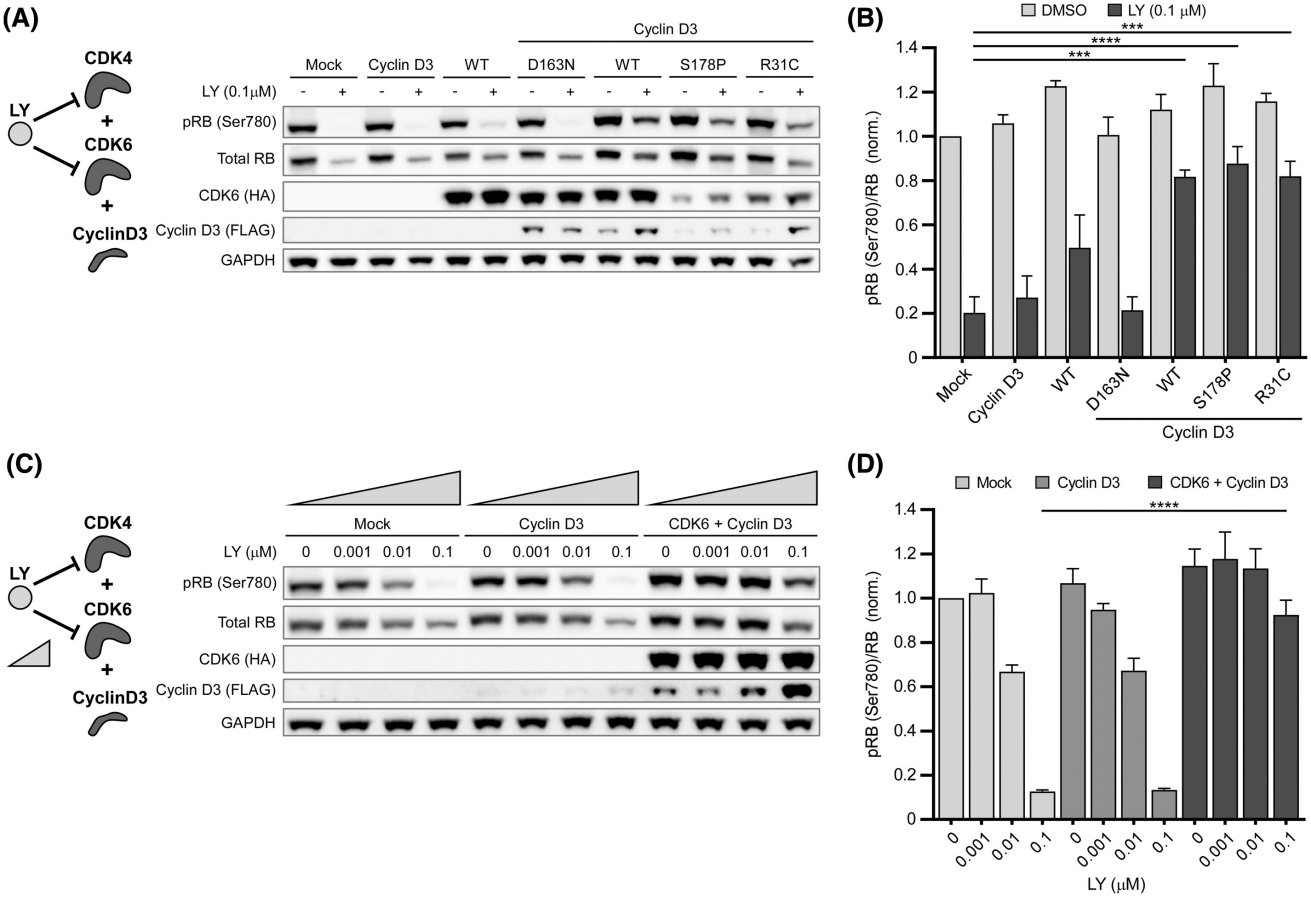
Kinase inhibition with LY reveals CDK6 activity. (A) Immunoblot analysis of pRB in MCF‐7 cells expressing CDK6 and its variants when co‐transfected with Cyclin D3 and treated with LY or DMSO. (B) Densitometry analysis of data shown in (A) expressed as a ratio of pRB to total RB. (C) Immunoblot analysis of pRB in MCF‐7 cells expressing CDK6 and Cyclin D3, treated with increasing concentrations of LY. (D) Densitometry analysis of data shown in (C) expressed as a ratio of pRB to total RB. (A and C) Representative experiments are shown. (B and D) One‐way ANOVA test with Dunnett's method correction compared with mock‐transfected LY‐treated control. (B) *n* = 4 and (D) *n* = 3. Data are mean ± SEM. ***p ≤ .001, ****p ≤ .0001.

Abemaciclib (LY2835219) (LY) is an inhibitor of CDK4 and CDK6 with similar half maximal concentrations (IC_50_ 2.0 and 9.9 nM).^47^, ^48^ Previous studies have shown that treatment of MCF‐7 cells with LY resulted in the emergence of drug resistance. This resistance was attributed to amplified CDK6 levels resulting in altered cellular inhibitor sensitivity.^27^, ^43^, ^44^, ^49^ Based on these observations, we hypothesized that application of LY may reduce background pRB levels in MCF‐7 cells and that consequently overexpressed CDK6 may become functionally detectable. We first treated the cells with 0.1 μM LY as this dose was previously shown to be sufficient for inhibition of pRB signals.^27^ In mock‐transfected cells this resulted in an almost complete reduction of RB phosphorylation (Figure 4A,B; this is attributed to CDK4 inhibition as CDK4 levels were not altered upon LY addition, Figure S8). In agreement with previous work,^27^, ^43^, ^50^, ^51^, ^52^ a reduction in total RB amounts was also observed in the presence of LY and the origins of this reduction are currently poorly understood. We next tested cells transfected with Cyclin D3 or CDK6 alone, CDK6 co‐transfected with Cyclin D3, and three CDK6 variants (the loss‐of‐function variant D163N and above gain of function variants) also co‐transfected with Cyclin D3 (Figure 4A,B; CDK4 levels were not altered upon CDK6 overexpression, Figure S8). Strikingly, we found that in the presence of LY overexpression of CDK6 induced pRB levels by ~2.5‐fold. Furthermore, co‐expression of CDK6 with Cyclin D3 resulted in an even greater increase by ~4.1‐fold (*p* < .0001). Similar to our previous experiments, the S178P or R31C variants produced comparable effects to CDK6. The D163N variant as expected showed low pRB levels similar to control cells which demonstrates that effects of ectopic expression of CDK6/Cyclin D3 are kinase dependent and attributable to active CDK6. Albeit a single dose of LY delivered the desired outcome of isolating CDK6 activity from the CDK4 background, we additionally performed dose response curves (Figure 4C,D). While reduction of pRB levels at increasing concentrations was observed in mock‐transfected and Cyclin D3 overexpressing cells, these levels were almost sustained in cells transfected with CDK6/Cyclin D3. A similar selection phenomenon was also observed using ribociclib (LEE‐011) (RI) or palbociclib (PD‐0332991) (PB), which are CDK4/6 inhibitors that are chemically not related to LY^48^, ^53^ (Figure S9). Overall, these results demonstrate that CDK6 activity can be isolated in a convergent motif even in the absence of a specific inhibitor.

### 3.5 In situ assay of CDK6 variants and modulators

**FIGURE 5:**
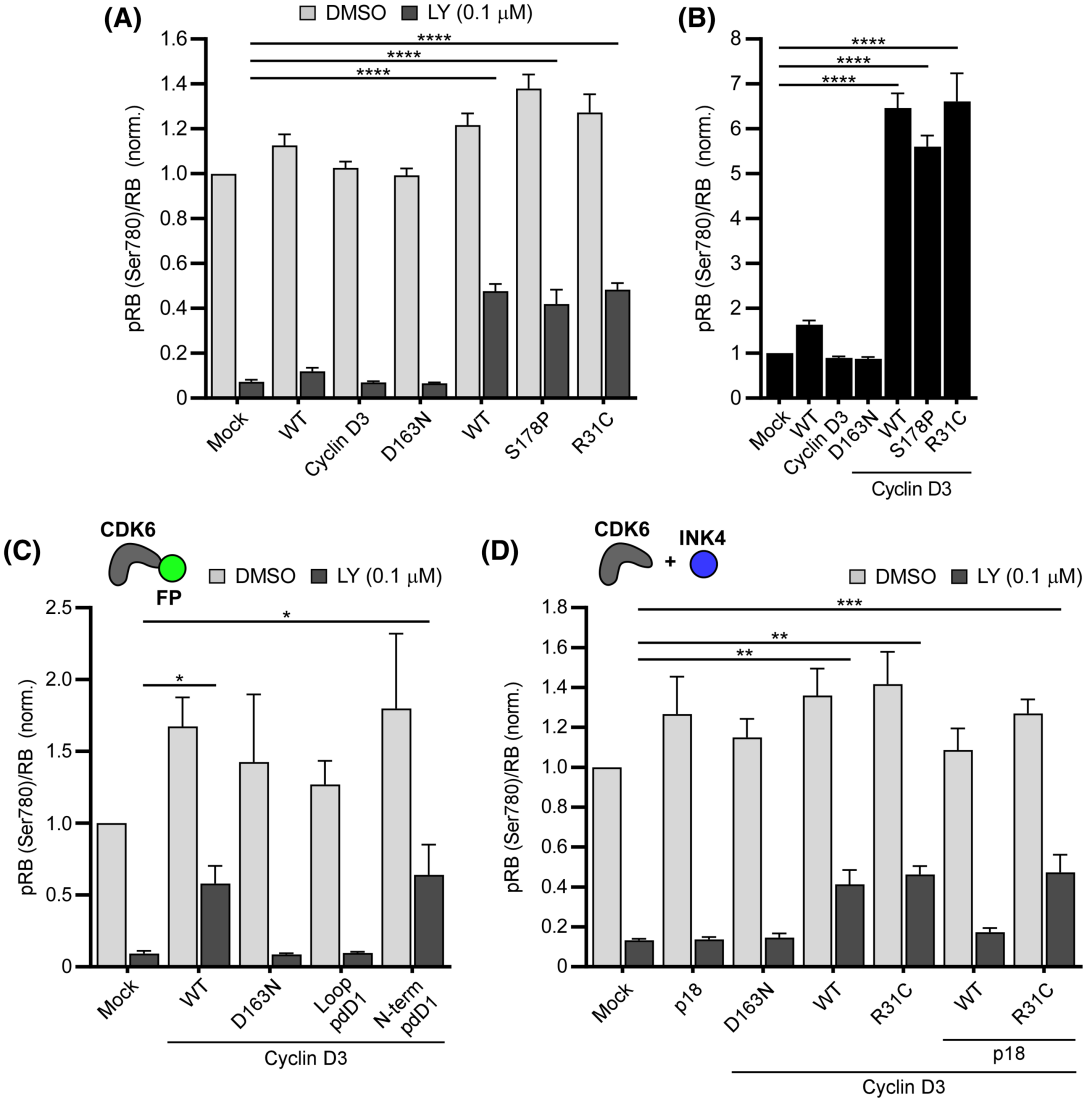
Energy transfer‐based assay of CDK6 variants and modulators. (A) Ratios of pRB to total RB for MCF‐7 cells expressing CDK6 and its variants co‐transfected with Cyclin D3. (B) LY‐treated conditions from (A) normalized to mock LY‐treated control. (C) Ratios for cells expressing CDK6 containing pdDronpa1 insertions within an internal loop (loop pdD1) or at the N‐terminus (N‐term pdD1). (D) Ratios for cells expressing p18^INK4c^ (p18) in combination with CDK6 or R31C variant and Cyclin D3. (A–D) One‐way ANOVA test with Dunnett's method correction compared with mock‐transfected LY‐treated control. *n* = 3. Data are mean ± SEM. *p ≤ .05, **p ≤ .01, ***p ≤ .001, ****p ≤ .0001.

We have demonstrated through immunoblotting that CDK6 activity can be quantified in cells using a combination of overexpression and CDK4/6 inhibition. We went on to test if this approach can be applied in a high‐throughput compatible platform and in the exploration of the function of engineered CDK6 variants. We employed an energy transfer‐based assay to detect pRB (Ser780) and total RB levels. This assay was chosen as it can be used with a greater efficiency than immunoblotting. We first tested the same conditions as above and observed similar results. CDK6 and activating mutations displayed the greatest fold increases in pRB levels specifically in the presence of LY (e.g., a >6‐fold difference in pRB levels compared to mock transfected inhibitor treated cells; Figure 5A,B). Also, as observed earlier, CDK6 alone only showed a minor increase and the two activating mutations overall behaved comparably.

Given the efficiency of this method, we applied it to study CDK6 function, inhibition, and regulation. We first examined genetically engineered versions of CDK6. Fluorescent proteins are commonly fused to enzymes as reporters of cellular levels/location or as functional regulators.^54^, ^55^, ^56^ These modifications can, however, be associated with a negative impact on protein function.^57^, ^58^ We introduced sequences encoding the photoactive pdDronpa1 protein^55^ at two separate sites of CDK6 to examine if insertion at either site alters activity. pdDronpa1 is a GFP‐like protein domain that has been previously used to generate photoswitchable (ps) kinases. Ps kinases, such as psCDK5, contain pdDronpa1 fused to the N‐terminus and in an internal loop.^55^, ^57^ We introduced pdDronpa1 at the N‐terminus of CDK6 (N‐term pdD1) as well as into an internal loop (loop pdD1; located between helices α_F_ and α_G_, homologous to insertion in psCDK5,^55^ Figure S10) as two separate constructs. We found that the N‐terminal insertion was well tolerated; however, insertion into the internal loop completely abolished activity (Figure 5C). Lastly, we explored the effects of protein modulators of CDK4/6 function by co‐transfecting the INK4 inhibitor protein p18^INK4c^ (p18). INK4 proteins are known to specifically inhibit and modulate CDK4/6 activity by disrupting the interaction with cyclin.^13^, ^59^ We transfected p18 alone or in combination with CDK6/Cyclin D3 or CDK6‐R31C/Cyclin D3. The R31C variant was included as it has previously been shown to reduce binding of INK4 proteins to CDK6.^45^ Indeed, we found that p18 was able to effectively inhibit CDK6 but unable to inhibit the R31C variant (Figure 5D). Overall, these findings establish an assay to test genetically modified CDK6 proteins and the effect of CDK6 modulators on kinase activity.

## 4 DISCUSSION

The organization of biological systems into networks permits the emergence of complex functions from a finite number of components and interactions. Kinase‐substrate interactions have been previously analyzed toward inference of the global network and targeted perturbation of disease‐related network elements. The objectives of our study were reductionist as we focused on local motifs and in situ assays. Specifically, we addressed three questions through a combination of bioinformatics and experiments: How common are cKSRs in experimentally validated Ser/Thr and Tyr phosphorylation events? What are the most common motif and family subgroup topologies? And, for the prototypical convergent CDK4/6 kinase pair, how can an individual kinase in the motif be studied experimentally?

To answer the first two questions, we systematically mapped human phosphorylation sites from the comprehensive PSP. Information in the experimentally validated PSP reflects on a range of factors that govern specificity in the phosphorylation network, such as evolved substrate recognition capabilities or protein localization in subcellular compartments or on adaptor proteins.^60^, ^61^ We found that a large number of human substrates are phosphorylated by more than one kinase. Furthermore, a majority of human kinases participate in cKSRs, and for both Ser/Thr and Tyr kinases a wide range of interaction topologies was observed. Finally, in many cases the kinases that act on a common substrate are from the same family subgroup and co‐expressed in a large fraction of analyzed cell models, which was assessed using both transcriptomic and proteomic datasets. These data collectively point to an abundance of convergent phosphorylation interactions that have the potential to drive multi‐site phosphorylation events (associated with amplification, crosstalk, and feedback) or redundant phosphorylation events (attributed as a source of diversity and robustness). It is important to discuss this work in the context of other kinome‐wide analysis methods. Our analysis is novel and complementary to “kinase‐substrate enrichment analysis” (KSEA),^62^ which exploits known KSRs to quantify the strength of these interactions in samples exposed to multiple experimental conditions. It is also complementary to prediction methods, such as “inference of kinase activities from phosphoproteomics” (IKAP) or “kinase activity analysis” (KAA),^63^, ^64^ that aim to predict KSRs from high‐throughput MS data, which is an active area of research, including in breast cancer.^65^ The objective of our work was to identify the fundamental properties of cKSRs as a basis for further sequence and coregulation analysis,^60^, ^66^ or for identification of kinases and substrates involved in relevant network motifs, as demonstrated for reciprocal phosphorylation events. We have focused on experimentally validated KSRs, including a separate analysis of a subset of KSRs that have been documented in multiple reports, to provide robust outcomes. The observation that related kinases phosphorylate substrates has here motivated the development of the experimental strategy that does not rely on kinase‐specific inhibitors.

To answer the third question, we studied the convergent CDK4/6 pair under conditions of kinase inhibition. ATP‐competitive CDK4/6 inhibitors have led to major improvements in the survival of breast cancer patients. Resistance has emerged in the patient population and, albeit studied intensively, no resistance inducing kinase mutations or substrate protein mutations have been observed. In turn, resistance was attributed to upregulation of CDK6 levels.^27^, ^43^, ^44^, ^49^ We hypothesized that the interplay of expression level and drug sensitivity can also be explored to design an in situ assay for CDK6. We performed these experiments in cells expressing high levels of CDK4 to provide a test bed in which this input entirely masked effects of initial CDK6 overexpression. Ultimately, this experimental strategy alloweded quantifying the activity of genetically modified CDK6 kinases and inhibitory accessory proteins in living cells. The emerging assay complements existing approaches to CDK6 that were in conducted in vitro.^32^, ^34^, ^43^ It also opens the door to more rapid protein engineering approaches that require quantification of cellular CDK6 activity. For instance, we showed that unlike CDK5,^55^ CDK6 is not amenable to internal pDronpa1 modification. The ability to perform these experiments with inhibitors from different chemical classes (as demonstrated here for LY, RI and PB) allows separating off‐target effects (e.g., from inhibition of upstream kinases). Several potential mechanisms for inhibitor resistance in CDK6‐amplified cells have been proposed. In the first mechanism, kinases at elevated protein levels are not efficiently inhibited at tuned drug concentrations, and consequently differential signals are recorded between cells with low (e.g., WT cells) or high CDK6 levels (e.g., CDK6 overexpressing cells). In a second mechanism, resistance of CDK6 to inhibitors may be modulated by accessory proteins.^43^ Our data suggest that the first mechanism is at play here as we observed a canonical inhibitory function of p18 in the presence of LY leading to reduced pRB levels. It is important to consider the biological context of kinase function when assessing convergence. For instance, in our case co‐overexpression of Cyclin D3 was required for a maximal effect, and notably D‐cyclins have also been found to be upregulated in expression upon exposure to CDK4/6 inhibitors.^27^, ^52^

Overall, we here analyzed phosphorylation maps using a convergence‐centric approach and, in a prototypical experimental system, dissected convergence. Many diverse cKSRs were observed despite the well‐documented bias of experimental phosphorylation datasets toward well‐studied kinases.^5^, ^8^, ^67^ Our experimental result was achieved even in the absence of kinase‐specific inhibitors (e.g., an inhibitor specific for CDK6 over CDK4) or knock‐out cell models of the individual converging kinases. Similar limitations may apply to many other scenarios involving convergent kinases. We developed a cellular assay for CDK6 that is desirable as experiments in cells may reflect on a complex natural environment of enzymes and an impact of cellular environment on drug properties. Quite likely redundancy and convergence also exist in other post‐translation modification networks, and our work may provide clues as to how to analyze these emerging phenomena.

## AUTHOR CONTRIBUTIONS

Christina Gangemi and Harald Janovjak were involved in conceptualization, funding acquisition, visualization, and writing—original draft. Rahkesh T. Sabapathy, and Harald Janovjak were involved in formal analysis. Christina Gangemi, Rahkesh T. Sabapathy, and Harald Janovjak were involved in methodology and data curation. Harald Janovjak was involved in project administration, supervision, and writing—review and editing. Christina Gangemi was involved in investigation.

## DISCLOSURES

The authors declare no competing interests.

## Supporting information


Data S1

## Data Availability

The data that support the findings of this study are available in the methods and/or supplementary material of this article. Software/code is available online (https://github.com/janovjaklab/convergent_kinases).
