# Supplementary material for: CDK6 activity in a recurring convergent kinase network motif
Source: FASEB J. 2023 Mar 8;37(4):e22845. doi: 10.1096/fj.202201344R (PMC11977600; doi:10.1096/fj.202201344R)
Supplement: Supplementary file 1 — Data S1 [file FSB2-37-e22845-s001.zip › FSB2_22845_202201344R-sup-0003-SI_Text-S02.pdf]

Gangemi *et al.* - Supplementary Information

Supplementary Tables S1 to S12: Please see separate Supplementary Data (Excel format).

Supplementary Table S13: Reciprocal phosphorylation loops. Loops of the topology shown in Supplementary Fig. S1 were identified in the cKSR data and literature searched for prior mention (see Methods for details).

Kinase A	Kinase B	Reaction A to B	Reaction B to A	Prior mention
Abl	MST1	Tyr	Ser/Thr	N
Akt1	CDK2	Ser/Thr	Ser/Thr	N
Akt1	CK2A1	Ser/Thr	Ser/Thr	N
Akt1	GSK3A	Ser/Thr	Ser/Thr	N
Akt1	mTOR	Ser/Thr	Ser/Thr	Y
Akt1	PIKFYE	Ser/Thr	Ser/Thr	N
Arg	PDGFRB	Tyr	Tyr	N
ASK1	PDK1	Ser/Thr	Ser/Thr	Y
Btk	ITK	Tyr	Tyr	Y
Btk	TEC	Tyr	Tyr	Y
CDK1	CDK7	Ser/Thr	Ser/Thr	N
CDK1	Chk1	Ser/Thr	Ser/Thr	Y
CDK1	CK2A1	Ser/Thr	Ser/Thr	N
CDK1	Wee1	Ser/Thr	Tyr	Y
CDK2	CDK7	Ser/Thr	Ser/Thr and Tyr	Y
Chk2	TTK	Ser/Thr	Ser/Thr	N
CK2A1	ERK2	Ser/Thr	Ser/Thr	N
EGFR	ERK1	Ser/Thr and Tyr	Ser/Thr	Y
EGFR	ERK2	Ser/Thr and Tyr	Ser/Thr	Y
ERK1	Lck	Ser/Thr	Tyr	Y
ERK1	MEK1	Ser/Thr	Ser/Thr and Tyr	Y
ERK2	MEK1	Ser/Thr	Ser/Thr and Tyr	Y
FAK	Ret	Tyr	Tyr	Y
GSK3B	Src	Ser/Thr and Tyr	Tyr	N
ITK	TEC	Tyr	Tyr	Y
MEK1	RAF1	Ser/Thr	Ser/Thr	Y
mTOR	p70S6K	Ser/Thr	Ser/Thr	Y
PKCD	Src	Ser/Thr	Tyr	Y

Supplementary Table S14: Protein sequences of WT CDK6 and engineered CDK6 variants.
Color code: Start codon. HA tag. WT human CDK6. Linkers. pdDronpa1.

CDK6	MYPYDVPDYAEKDGLCRADQQYECVAEIGEGAYGKVFKARDLKNGGRFVALKRVRVQTGEEGMPLSTIREVAV<br>LRHLETFEHPNVVRLFDVCTVSRTDRETKLTLVFEHVDQDLTTYLDKVPEPGVPTETIKDMMFQLLRGLDFLH<br>SHRVVHRDLKPQNILVTSSGQIKLADFGLARIYSFQMALTSVVVTLWYRAPEVLLQSSYATPVDLWSVGCIFA<br>EMFRRKPLFRGSSDVDQLGKILDVIGLPGEEDWPRDVALPRQAFHSKSAQPIEKFVTDIDELGKDLLLKCLTF<br>NPAKRISAYSALSHPYFQDLERCKENLDSHLPPSQNTSELNTA
pdDronpa1-CDK6 (N-term pdD1)	MYPYDVPDYASVIKPDMKIKLRMEGAVNGHPFAIEGVGLGKPFEGKQSIDLKVKEGGPLPFAYDILTTAFCYG<br>NRVFAKYPENIVDYFKQSFPEGYSWERSMNYEDGGICNATNDITLDGDCYIYEIRFRGTNFPANGPVMQKRTV<br>KWEPSTENLYVRDGVLKGDVVMALSLEGGGHYRCDFKTTYKAKKVVQLPDYHFVDHHIEIKSHDKDYSNVNLH<br>EHAEAHSELPRQAKGLKGSGEKDGLCRADQQYECVAEIGEGAYGKVFKARDLKNGGRFVALKRVRVQTGEEGM<br>PLSTIREVAVLRHLETFEHPNVVRLFDVCTVSRTDRETKLTLVFEHVDQDLTTYLDKVPEPGVPTETIKDMMF<br>QLLRGLDFLHSHRVVHRDLKPQNILVTSSGQIKLADFGLARIYSFQMALTSVVVTLWYRAPEVLLQSSYATPV<br>DLWSVGCIFAEMFRRKPLFRGSSDVDQLGKILDVIGLPGEEDWPRDVALPRQAFHSKSAQPIEKFVTDIDELG<br>KDLLLKCLTFNPAKRISAYSALSHPYFQDLERCKENLDSHLPPSQNTSELNTA
CDK6-internal-pdDronpa1 (loop pdD1)	MYPYDVPDYAEKDGLCRADQQYECVAEIGEGAYGKVFKARDLKNGGRFVALKRVRVQTGEEGMPLSTIREVAV<br>LRHLETFEHPNVVRLFDVCTVSRTDRETKLTLVFEHVDQDLTTYLDKVPEPGVPTETIKDMMFQLLRGLDFLH<br>SHRVVHRDLKPQNILVTSSGQIKLADFGLARIYSFQMALTSVVVTLWYRAPEVLLQSSYATPVDLWSVGCIFA<br>EMFRRKPLFRGTGGSVIKPDMKIKLRMEGAVNGHPFAIEGVGLGKPFEGKQSIDLKVKEGGPLPFAYDILTTA<br>FCYGNRVFAKYPENIVDYFKQSFPEGYSWERSMNYEDGGICNATNDITLDGDCYIYEIRFRGTNFPANGPVMQ<br>KRTVKWEPSTENLYVRDGVLKGDVVMALSLEGGGHYRCDFKTTYKAKKVVQLPDYHFVDHHIEIKSHDKDYSN<br>VNLHEHAEAHSELPRQAKRTSSDVDQLGKILDVIGLPGEEDWPRDVALPRQAFHSKSAQPIEKFVTDIDELGK<br>DLLLKCLTFNPAKRISAYSALSHPYFQDLERCKENLDSHLPPSQNTSELNTA

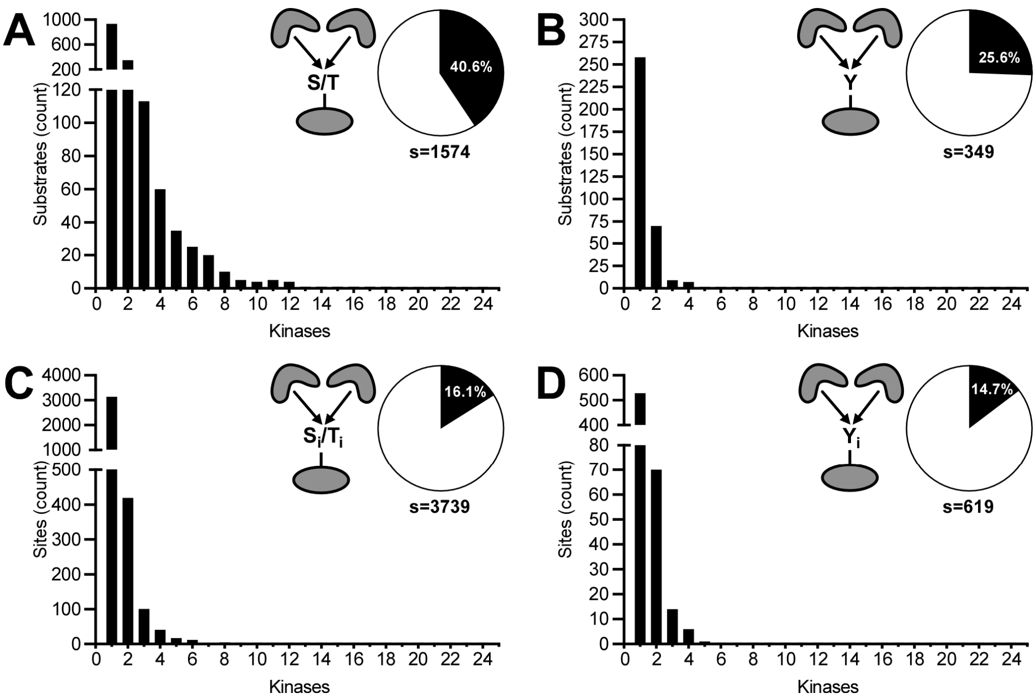

Supplementary Figure S1: Substrate-centric analysis of cKSRs considering only phosphosites that were identified in multiple reports (see Methods for details). Distribution histograms of general and site-specific cKSRs for Ser/Thr phosphorylation (**A and C**) and Tyr phosphorylation (**B and D**). Bars indicate how many substrates are phosphorylated by the indicated number of kinases. Pie charts indicate the percentage of substrates that are phosphorylated by ≥two kinases and the total number of analyzed substrates (s).

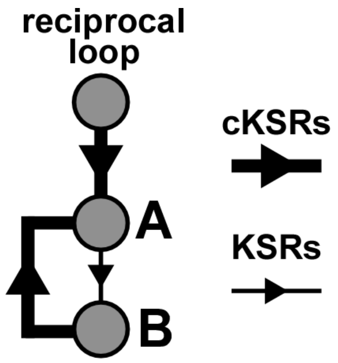

Supplementary Figure S2: Topology of reciprocal phosphorylation loops. The general cKSR dataset was analyzed to identify all kinases A that receive two inputs and whose substrates B are phosphorylating these kinases reciprocally. The identified kinase pairs are listed in Supplementary Table S13.

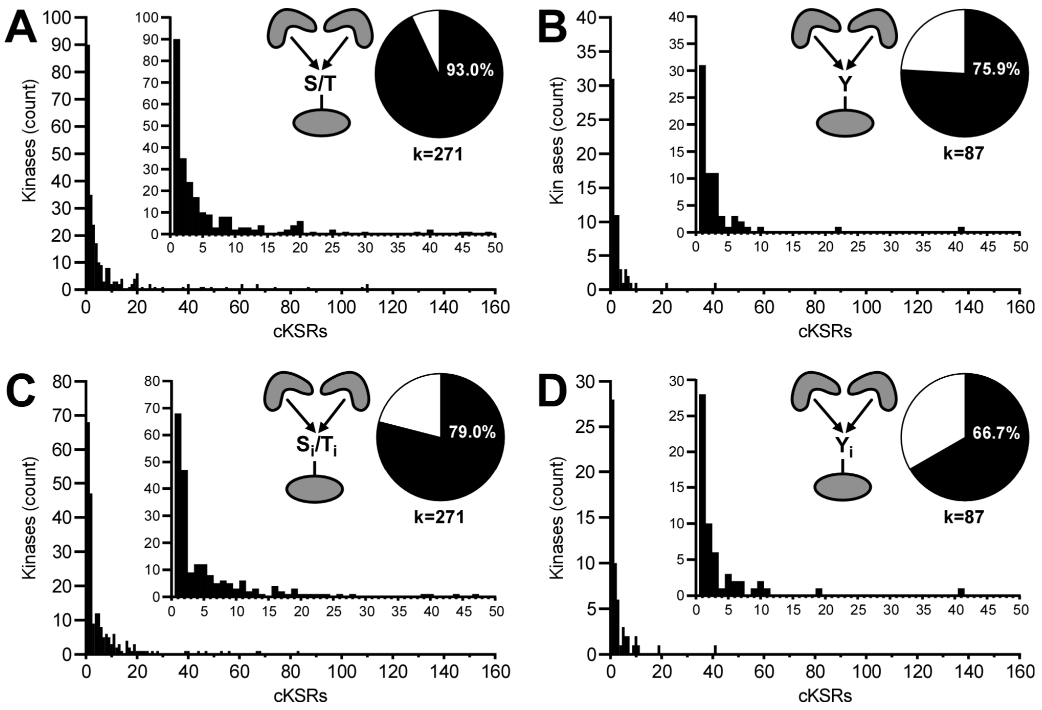

Supplementary Figure S3: Kinase-centric analysis of cKSRs considering only phosphosites that were identified in multiple reports (see Methods for details). Distribution histograms for Ser/Thr kinases (**A and C**) and Tyr kinases (**B and D**) in general cKSRs (**A and B**) and site-specific cKSRs (**C and D**). Bars indicate how many kinases phosphorylate the indicated number of substrates. Pie charts indicate the percentage of kinases that participate in general cKSRs and the total number of kinases (k).

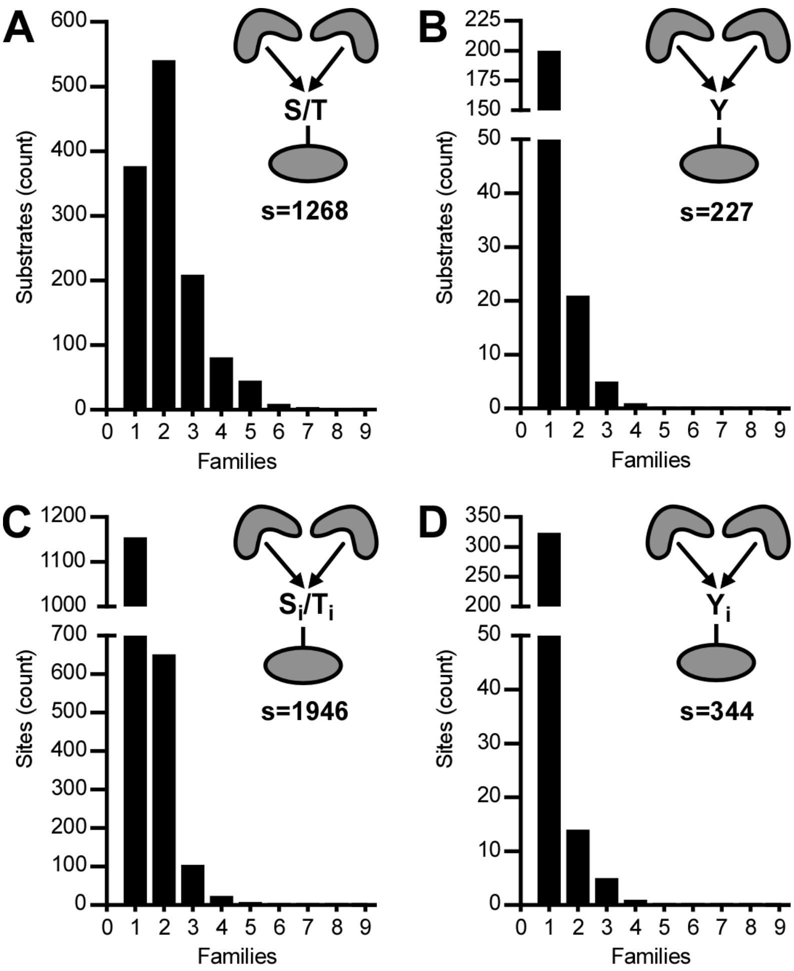

Supplementary Figure S4: Distribution histograms of kinase families participating in convergent Ser/Thr phosphorylation (A, C) and Tyr phosphorylation (B, D). Bars indicate the number of substrates (A, B) or sites (C, D) that are phosphorylated by members of the indicated number of kinase families. s: Analyzed number of substrates or sites.

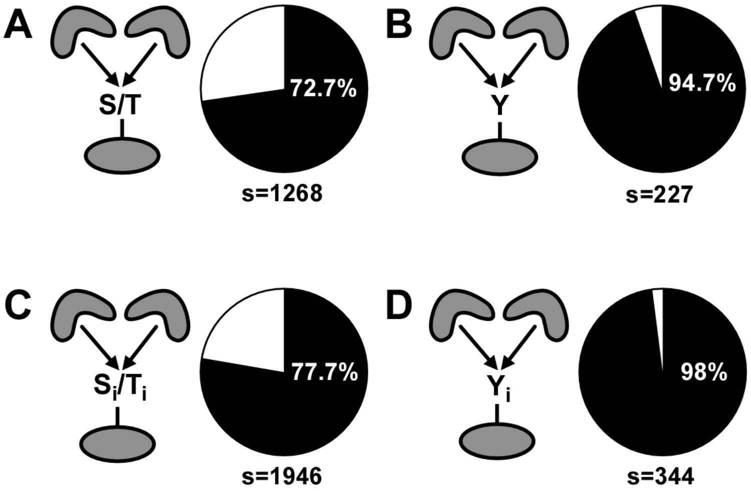

Supplementary Figure S5: Occurrence of phosphorylation events that include ≥two kinases from the same family subgroup. Pie charts indicating the percentage of substrates that are phosphorylated by ≥two kinases from at least one family subgroup, for general and site-specific Ser/Thr phosphorylation (A, C) and general and site-specific Tyr phosphorylation (B,D). s: Analyzed number of substrates or sites.

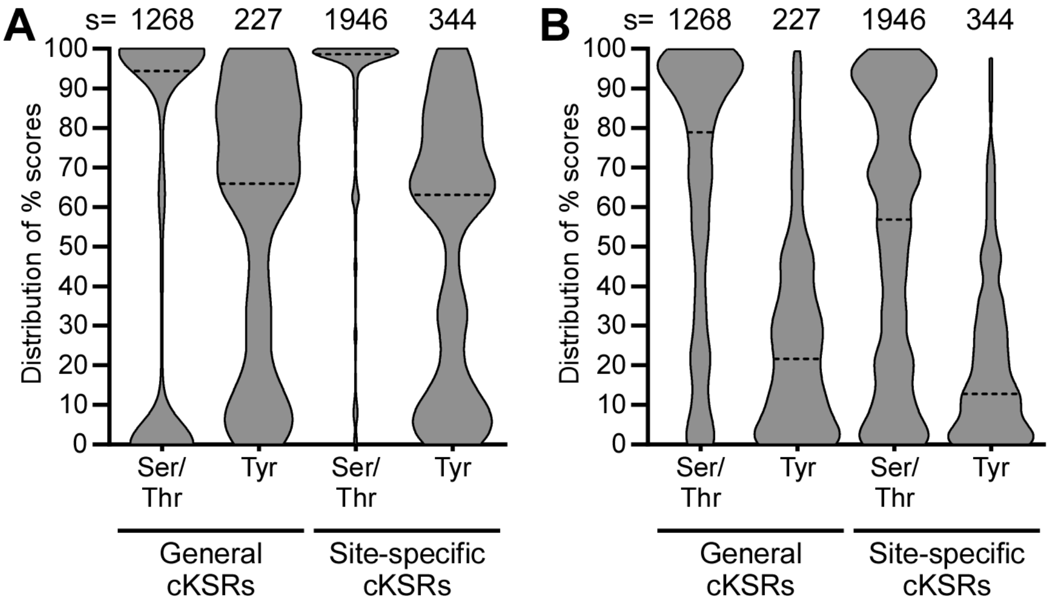

Supplementary Figure S6: Co-expression analysis at different TPM thresholds. (**A**) TPM threshold of 5. (**B**) TPM threshold of 20. Co-expression scores and median (dashed line) are defined as in Fig. 2. s: Analyzed number of substrates or sites.

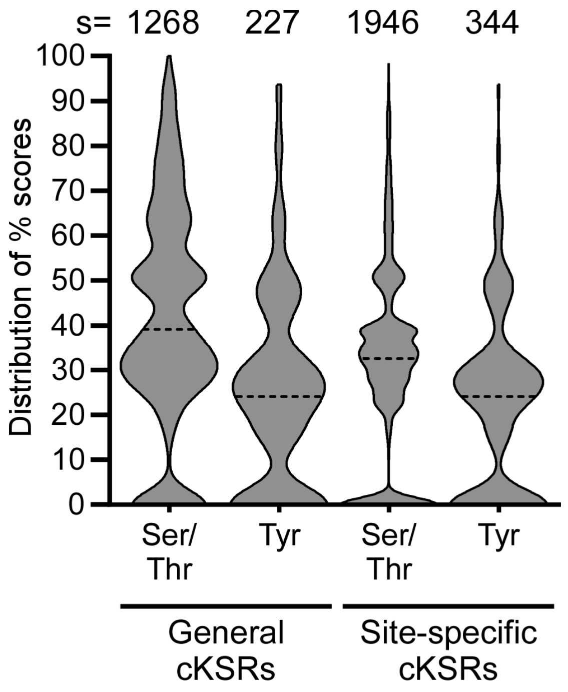

Supplementary Figure S7: Co-expression analysis based on proteomics data. Co-expression scores and median (dashed line) are defined as in Fig. 2. s: Analyzed number of substrates or sites.

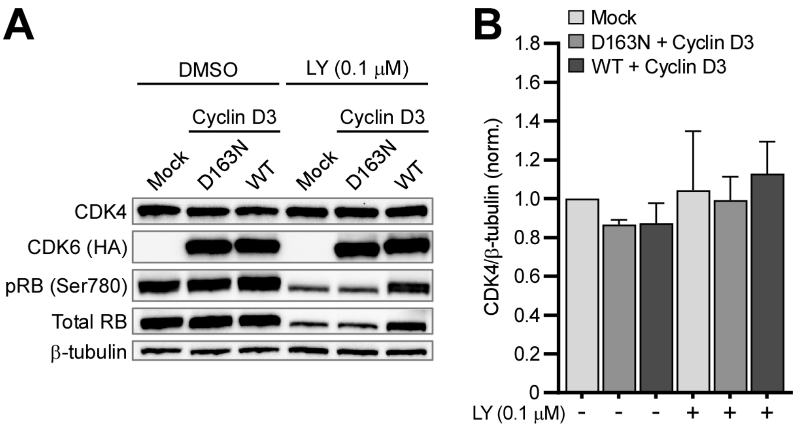

Supplementary Figure S8: CDK4 levels upon CDK6 overexpression and LY treatment. (**A**) Representative immunoblot of CDK4 in MCF-7 cells expressing WT CDK6 or loss-of-function variant D163N with Cyclin D3 and treated with LY where indicated. (**B**) Densitometry analysis of data shown in (A) expressed as a ratio of CDK4 to β-tubulin loading control. n=3. Data are mean ± SEM. One-way ANOVA test with Dunnett's method correction compared with mock-transfected DMSO control: No significance was detected.

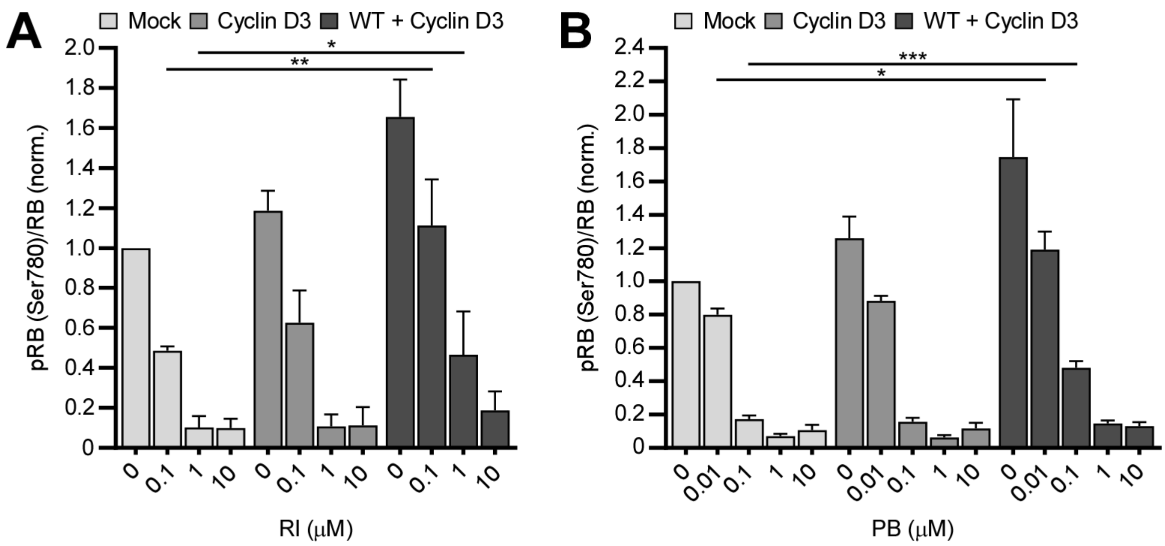

Supplementary Figure S9: Kinase inhibition with RI or PB reveals CDK6 activity. Energy transfer-based assays to detect ratios of pRB to total RB in MCF-7 cells expressing CDK6 and Cyclin D3 treated with increasing concentrations of RI (**A**) or PB (**B**). One-way ANOVA test with Dunnett's method correction compared with mock-transfected LY-treated controls. n=3. Data are mean ± SEM. *: $p \leq .05$, **: $p \leq .01$, ***: $p \leq .001$.

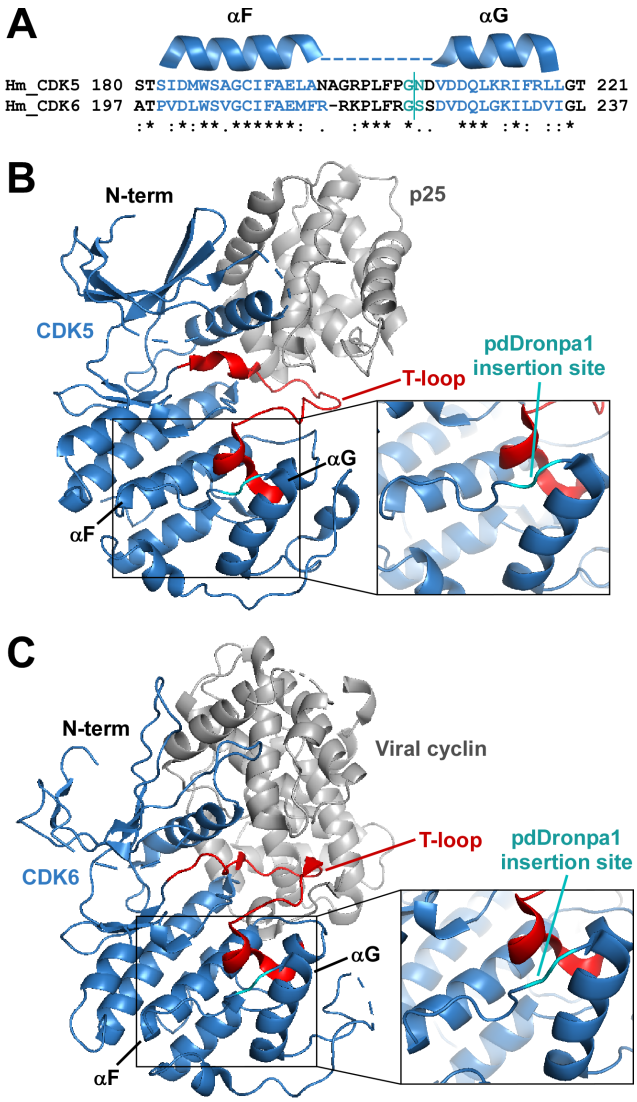

Supplementary Figure S10: Analogous insertion sites of pDronpa1 in CDK5 and CDK6. (**A**) Protein sequence alignment of human CDK5 and CDK6. pdDronpa1 internal loop insertion site highlighted in cyan (for CDK5, the insertion is equivalent to that of psCDK5 (Zhou et al., 2017)). (**B** and **C**) CDK5-p25 and CDK6-viral cyclin complex structures with the insertion sites highlighted (PDB-IDs: 1H4L for CDK5 and 1JOW for CDK6).

References

Zhou, X.X., Fan, L.Z., Li, P., Shen, K., and Lin, M.Z. (2017). Optical control of cell signaling by single-chain photoswitchable kinases. Science *355*, 836-842.
